# Frequency and Factors of Sleep Paralysis Among Medical Students of Karachi

**DOI:** 10.7759/cureus.41722

**Published:** 2023-07-11

**Authors:** Muhammad Ali Muzammil, Abdul-Rehman Syed, Muhammad Haris Farooq, Shaheer Ahmed, Muhammad Hassam Qazi, Tirath Patel, Mahima Khatri, Mohammad Uzair Zaman, Taha Nadeem, Fatima Tanveer, Umesh Kumar, Giustino Varrassi, Abdul Ahad Shah

**Affiliations:** 1 Internal Medicine, Dow University of Health Sciences, Karachi, PAK; 2 Medicine, Dow University of Health Sciences, Karachi, PAK; 3 Medicine, Karachi Medical and Dental College, Karachi, PAK; 4 Medicine, American University of Antigua, St. John, ATG; 5 Medicine and Surgery, Dow University of Health Sciences, Karachi, PAK; 6 Medicine, Bacha Khan Medical College, Mardan, PAK; 7 Medicine, Allama Iqbal Medical College, Lahore, PAK; 8 Pain Medicine, Paolo Procacci Foundation, Rome, ITA

**Keywords:** sleep social jetlag, rem sleep, rem sleep behavior disorder, hypertension, seizure, electroencephalogram, neurological, narcolepsy, psychiatric

## Abstract

Introduction: Sleep paralysis is a prevalent phenomenon characterized by suffocation, immobility, and hallucinations. Its causes remain unknown, although the neurotransmitter imbalance is suggested as a potential factor. This condition is closely associated with hallucinations and a sense of intrusion, often observed in patients with narcolepsy, hypertension, and seizures.

Methods: A cross-sectional study was conducted in various medical colleges in Karachi, involving 297 participants aged 18 to 30 years. The participants were divided into groups based on gender and year of study. They were surveyed about the frequency of sleep paralysis episodes, their beliefs about the phenomenon, sleep routines, and academic impacts.

Results: Among the respondents, a significant number of females (n=209, 70.3%) reported experiencing sleep paralysis. The overall mean age was 20±2.0 years. Correlation analysis revealed an insignificant relationship between depression and mental anxiety (p=0.147). Similarly, no significant association was found when comparing the occurrence of sleep paralysis (p=0.16). However, a notable finding was the significant link between sleep paralysis and its impact on academics (p=0.043).

Conclusion: This study highlighted the frequency of sleep paralysis among medical students, particularly among females. Furthermore, it emphasizes the diverse beliefs held by individuals regarding these frightening episodes. To address this neglected issue, it is essential to conduct awareness sessions aimed at understanding and alleviating sleep paralysis in individuals' lives.

## Introduction

Sleep paralysis is a paralytic episode experienced by an individual, typically at the onset of sleep or before waking up. It has been found that sleep paralysis occurs during the rapid eye movement (REM) stage of sleep, in which the person’s vitals, such as respiratory rate, pulse, and blood pressure, elevates. It has been found that during REM, the electroencephalogram (EEG) shows some unusual waves and frequencies similar to the EEG of a person who is awake [1].

During sleep paralysis, the person cannot move their limbs and extremities, which may result in chest tightness and suffocation. Despite this fact, it has been found that individuals cannot open their eyes during the episodes and are conscious of their surroundings [2]. The exact cause of sleep paralysis is still a mystery. But in some studies, it is found that during sleep paralysis, glycine and GABA level increases which inhibit motor neurons [3]. Many episodes are related to terror and deep anguish; in some cases, it only increases stress and discomfort. Most incidences are found in early adulthood, while the remaining may continue throughout life [4]. Social life and psychological state of mind are profoundly affected by sleep-paralytic episodes. Sleep paralysis shows a close association with narcolepsy, hypertension, and seizures, and it may be secondary to lack of sleep, jet lag, sleep disturbances, and extended shifts [5]. It has been reported that the rate of sleep paralysis is 49% in narcoleptics, 18% among hyper somnolent patients, and just 5% among well-screened health control individuals [6]. Sleep paralysis has shown a close link with hallucination, where an individual experiences a sense of intruder that may or may not be present at that time. It is also linked with incubus hallucination, a frightening hallucination that makes the person feel strangled, hovering to death, or that someone may be sitting on his chest [7]. Fear is a factor that is considered consistent with the intruder and incubus experiences with the hypothesis of the mechanisms that overview the reactions to threat and assault [8].

Throughout history, different cultures had different views related to sleep paralysis. For example, Canadian Eskimos had a belief in spells of shamans that could paralyze the ability to move and could stimulate hallucinations. Japanese believe that sleep paralysis occurs due to a vengeful spirit that suffocates his enemies during sleeping. Nigerians believe that a female demon possesses the individual and paralyzes their body [9]. The sooner the incidence of sleep paralysis emerges in an individual, the more frequent its episodes are [3,10]. 

The primary objective of our research was to study the frequency of sleep paralysis episodes among medical students, and the secondary objective was to investigate its relation with its contributing factor.

## Materials and methods

This cross-sectional study was conducted with a proportionate stratified random sampling technique from March 2022 to April 2023 among different Medical Colleges in Karachi. All participants who showed up were interrogated using a pre-designed questionnaire and enrolled only after taking informed and signed consent. The interview was conducted by two trained interviewer who was fluent in English. The study was comprised of 315 participants.

Eighteen of these 315 participants didn't complete the form and left the interview. Consequently, by using openepi.com the cooperation rate was 95%. Only those participants included in the study who gave complete data, individuals between 18 and 30 years of age, students of MBBS, BDS, DPT, Pharm-D, or any other medical field, studying in their respective college for at least six months and have experienced sleep paralysis once in their life were included in the study. Conversely, the study excluded individuals who did not meet the age criteria or were not enrolled in medical colleges. Additionally, those who were unwilling to participate or provide consent were excluded from the study. The implementation of these criteria ensured a representative sample of medical students within the specified age range, facilitating the investigation into the frequency and factors associated with sleep paralysis in this particular population. A pilot study was conducted to assess the questionnaire's validity and to correct errors in the data collection technique. All participants were subjected to sleep paralysis, and different symptoms of sleep paralysis were found.

Our questionnaire was designed to investigate whether medical students had sleep paralysis and whether stress was the leading role player. Numerous questions were asked to examine participants' sleep routines, frequency of sleep paralysis, and its probable causes. To have a clear idea, further specific questions were asked, such as the time at night when paralytic dreams were experienced, any prior traumatic experience, significant occurrences on weekdays or weekends, and sleeping positions when they awoke from a paralytic episode. We also devised a question allowing the participants to mark multiple responses for their paralytic attacks, if any. And finally, they were asked about their stress level on a scale of 1 to 10 to reconfirm further that despite numerous reasons, stress itself had immense significance or not.

Data analysis

Data were entered in Microsoft Excel and analyzed using SPSS version 22 (Armonk, NY: IBM Corp.). The chi-square test was used to assess associations between categorical variables. The t-test and ANOVA were used to compare quantitative variables. Statistical significance was set at p<0.05.

## Results

The study was conducted among 297 mainstream students (n=209, 70.3% females) with a mean age of 20±2.0 years. Students from different medical colleges in Karachi filled out the questionnaire; the maximum number of responses were from second-year students (n=115, 38.46%). Among departments, most students were of MBBS (n=114, 38.12%). A parallel integer of students (n=69, 23.07%) participated from BDS and Doctorate of Pharmacy departments, followed by Doctorate of Physiotherapy students (n=47, 15.71%).

In Table 1, many participants (n=167, 56%) experienced moderate nature dreams, whereas a smaller portion (n=71, 22%) marked disturbed sleep patterns. Only a tapered number sleep comfortably (n=22, 8%). However, fewer undergraduates (n=37, 13%) experienced no dream, showing little relation when compared (p=0.071). An ample amount, specifically females, frequently experience nightmares (n=175, 83.7%) as compared to males (n=69, 78.4%). When episodes of nightmares per night were compared, estimated a significant correlation (p=0.002). A sizeable population (n=111, 35%) reported frequent attacks. On the contrary, (n=46) 16% experienced it infrequently while (n=71) 33% suffered rarely. Few of them (n=53, 19%) reported no nightmares.

**Table 1:** Gender-based analysis to assess the effect of gender on overall responses.

Questions	Response	Male	Female	p-Value
Have you ever experienced sleep paralysis?	Yes	48 (54.5%)	144 (68.9%)	0.001
	No	40 (45.5%)	65 (31.1%)	
If yes, how often?	At least one episode per week	8 (9.1%)	19(9.1%)	0.16
	More than one episode per week	5 (5.6)	32 (6.7%)	
	At least one episode per month	13 (14.8%)	34 (16.3%)	
	More than one episode per month	9 (4.4%)	2 (2.3%)	
	One episode per year	11 (12.5%)	33 (15.8%)	
What are the causes of sleep paralysis?	Witch crafting	37 (42.0%)	91 (43.5%)	0.048
	Drug abuse	57 (64.8%)	120 (57.4%)	
	Nightmares	25 (28.4%)	57 (27.3%)	
	Dying dreams	72 (81.9%)	150 (71.8%)	
	Paranormal activities	44 (50.0%)	76 (36.4%)	
	Flying dreams	47 (53.4%)	76 (36.4%)	
	Hypnopompic and hypnagogic imaginations	48 (54.5%)	142 (67.9%)	
Do you think sleep paralysis leads to anxiety?	Yes	73 (83.0%)	151 (72.2%)	0.147
	No	15 (17.0%)	58 (27.7%)	
Do you think people with sleep paralysis are reluctant to talk about this topic?	Yes	39 (44.3%)	105 (50.2%)	0.008
	No	49 (55.7%)	104 (49.8%)	
What did you experience during your sleep paralytic episodes?	Inability to move	39 (44.3%)	99 (47.4%)	0.045
	Inability to speak	39 (44.3%)	106 (50.7%)	
	Sense of suffocation	24 (27.3%)	57 (27.3%)	
	Unusual auditory or visual sensation	26 (29.5%)	98 (46.9%)	
	Attack preceded by terrifying dream or thought	14 (15.9%)	58 (27.8%)	
Do you have a family history or experience any of these conditions?	Automatic behavior	1 (1.1%)	5 (2.4%)	0.061
	Bipolar disorder	4 (4.5%)	12 (5.7%)	
	Hallucination	1 (1.1%)	4 (1.9%)	
	Non-restorative sleep	2 (2.3%)	11 (5.3%)	
	Nocturnal leg cramps.	4 (4.5%)	26 (12.4%)	
	Sleep apnea	0 (0.0%)	4 (1.9%)	
	Insomnia	8 (9.1%)	29 (13.9%)	
	Narcolepsy	1 (1.1%)	8 (3.8%)	
	None	66 (75.0)	110 (52.6%)	
How many hours do you sleep during weekdays?	4 h	8 (9.09%)	15 (7.2%)	0.01
	6 h	42 (47.7%)	105 (50.2%)	
	8 h	30 (34.1%)	69 (33.0%)	
	10 h	4 (4.5%)	15 (7.2%)	
	12 h	4 (4.5%)	5 (2.39%)	
How many hours do you sleep during the weekend?	4 h	2 (2.3%)	3 (1.4%)	0.069
	6 h	4 (4.5%)	12 (5.7%)	
	8 h	36 (40.9%)	74 (35.4%)	
	10 h	36 (40.9%)	86 (40.1%)	
	12 h	10 (11.4%)	34 (16.3%)	
How many hours of sleep do you require to feel fresh?	4 h	3 (3.4%)	12 (5.7%)	0.006
	6 h	15 (17.0%)	34 (16.3%)	
	8 h	45 (51.1%)	100 (47.8%)	
	10 h	13 (14.8%)	47 (22.5%)	
	12 h	12 (9.1%)	16 (7.7%)	
Do you experience any paralytic episodes during your daytime sleep?	Yes	10 (11.3%)	36 (17.2%)	0.084
	No	78 (88.6%)	173 (82.8%)	
At what age did you first experience sleep paralysis?	Less than 10 years	4 (4.5%)	8 (3.8%)	0.027
	11-14 years	8 (9.1%)	30 (14.4%)	
	15-20 years	29 (33.0%)	95 (45.5%)	
	>20 years	3 (3.4%)	11 (5.2%)	
	>25 years	2 (2.3%)	0 (0.0%)	
	>30 years	2 (2.3%)	0 (0.0%)	
At what time do u experience sleep paralysis?	Beginning of sleep	4 (4.5%)	15 (7.2%)	0.003
	Middle of the night	25 (28.4%)	71 (34.0%)	
	End of sleep	10 (11.4%)	30 (14.4%)	
	More prevalent during the first 2 h after sleep onset	7 (8.0%)	19 (9.1%)	
	At sleep offset	2 (2.3%)	5 (2.4%)	
Do you use mobile phone before bedtime?	Yes	80 (90.9%)	198 (94.7%)	0.007
	No	8 (9.0%)	11 (5.2%)	
What kind of online content do you use before bedtime?	Online magazines	0 (0.0%)	3 (1.4%)	0.095
	E-commerce	2 (2.2%)	5 (2.39)	
	Blogs	4 (4.5%)	6 (2.9%)	
	Portfolio	1 (1.1%)	3 (1.4%)	
	Social media	81 (92.0%)	192 (91.9%)	
What was your sleeping position when you experienced sleep paralysis?	Supine	23 (26.1%)	81 (38.8%)	0.036
	Prone	8 (9.1%)	10 (4.8%)	
	Left-side	7 (8.0%)	28 (13.4%)	
	Right-side	10 (11.4%)	25 (12.0%)	
What type of dream you usually experience?	Peaceful	9 (10.2%)	13 (6.2%)	0.071
	Disturbed	15 (17.0%)	56 (26.8%)	
	Moderate	50 (56.8%)	117 (56.0%)	
	None	14 (15.9%)	23 (11.0%)	
Have you experienced nightmares?	Yes	69 (78.4%)	175 (83.7%)	0.037
	No	19 (21.6%)	34 (16.3%)	
If yes, how often?	Always	1 (1.4%)	15 (8.5%)	0.002
	Sometime	26 (37.6%)	85 (48.5%)	
	Infrequently	14 (20.2%)	32 (18.2%)	
	Rarely	28 (40.5%)	43 (24.5%)	
Were you always in a state of stress during your sleep paralysis episodes?	Yes	17 (35.4%)	74 (51.3%)	0.023
	No	31 (64.5%)	70 (48.6%)	
If yes, what is the reason of your stress?	Financial problem	3 (17.6%)	10 (13.5%)	0.007
	Academic burden	9 (52.9%)	44 (59.4%)	
	Any traumatic experience	5 (29.5)	17 (22.9%)	
	Family Issues	0 (0.0%)	3 (4.2%)	
Do you think sleep paralysis has relation with your life events?	Yes	26 (54.1%)	73 (50.6%)	<0.001
	No	22 (45.9%)	71 (49.4%)	
Do you think sleep paralysis episodes affect your academics?	Yes	17 (35.4%)	46 (31.9%)	0.043
	No	31 (64.6%)	98 (68.1%)	

When questioned about an episode of sleep paralysis, responses showed a (p=0.004) significant association (Table 1). Females had sleep paralysis episodes (n=144, 68.9%) more than males (n=48, 54.5). Numerous participants (n=47, 16%) marked at least one per month, followed by one episode per year (n=44, 14%), one episode per week (n=27, 9.1%), and more than one episode per week (n=37, 6%) allied trivially (p=0.16). Furthermore, symptoms during sleep paralysis (p=0.045) were linked ominously to responses. Data analysis revealed that (n=145) 48% of individuals could not speak, and almost a similar populace (n=138, 46%) could not move during the paralytic episode. Other symptoms, such as a sense of suffocation (n=81, 27%), unusual auditory or visual sensation (n=124, 38%), and attack preceded by a terrifying dream of thought (n=72, 22%), were also noted.

A comparison of sleep paralysis and age of onset discovered (p=0.027) a powerful bond. In Table 1, the more significant part (n=176, 64%) doesn’t give an account of any sleep paralysis family history at all. Very few agreed upon experiencing one of the following conditions. A more significant part chose nocturnal leg cramps (n=30, 8%) and insomnia (n=37, 12%), while other disorders, such as automatic behavior (n=6, 2%), bipolar disorder (n=16, 5%), hallucination (n=5, 2%), non-restorative sleep (n=13, 4%), sleep apnea (n=4, 1.9%), and narcolepsy (n=9, 2%) marked by a minority, showing no significant association (p=0.061).

On analyzing total hours of sleep on weekdays with responses, only (n=147, 49%) slept 6 hours/day. The other half (n=99, 34%) reported eight hours/day sleep, while a small portion (n=22, 8%) had 4 and 10 hours (n=19, 6%) of sleep per day. However, for weekends the data compiled connects results insignificantly (p=0.069). The majority of participants (n=122, 41%) were likely to sleep 10 hours, while others slept for 6 hours (n=16, 5%) and 8 hours (n=110, 38%) only. Very few were found to (n=5, 2%) have 4 hours of sleep, and (n=44) 14% astoundingly slept for 12 hours. A loftier population (n=149, 52%) found sleeping 8 hours as enough time to feel fresh. A tranquil amount (n=49, 17%) considered 6 hours enough time to become energetic. In contrast, a small portion (n=15, 5%) felt 4 hours were sufficient to regain energy.

A significant relation was obtained (p=0.003) when compared with the time of the paralytic episode; an enormous amount experienced it in the middle of the night (n=96, 31%) whereas (n=40, 13%) at the end of sleep and during first two hours of sleep (n=26, 9%) episodes were also highlighted. Very few cases (n=19.6%) experience sleep paralysis at sleep onset. Paralytic incidents were also found in partakers during the daytime to bring about an insignificant outcome (p=0.084). A momentous relation (p=0.007) was established when compared with mobile usage before bedtime between females (n=198, 94.7%) and males (n=80, 90.9%). Many use social media websites (n=273, 92%), imparting insignificantly (p=0.095) to the responses. Sleeping positions also show a significant result (p=0.036) when equaled. The voluminous amount marked the supine position (n=104, 32%), while the lingering portion nominated left-sided (n=35, 11%) and right-sided (n=35, 12%) as the paralytic sleeping position. Least number of students (n=18, 7%) experience sleep paralysis in the prone position.

When evaluating for the likely cause of sleep paralysis, it favors (p=0.048) an inconsequential bond where the majority (n=222, 76.85%) consider dying dreams as the probable cause, followed by hypnopompic and hypnogogic imaginations (n=190, 61.2%). Drug abuse was also nominated (n=177, 61%) as a significant cause. Other causes such as (n=128, 42%) witch-crafting and paranormal activities (n=82, 120, 43%) ponder as minor causes.

The female population (n=74, 51.3%) likely attained a state of stress during paralytic episodes as compared to males (n=17, 35.4%), concluding significantly (p=0.023). However, a significant population denied being stressed during sleep paralysis (n=101, 56.55%). When the relationship between stress and sleep paralysis was assessed, they showed an insignificant association (p=0.007), mentioning academic burden as the main reason for anxiety.

Besides, (n=63, 34%) undergraduates deliberate sleep paralysis as a root of academic distortion while reasonable size (n=129, 66%) disagrees, commencing a significant correlation (p=0.043). Formerly, a correlation was established (p=0.147) that interrogated sleep paralysis with depression and mental anxiety, confining individuals to discuss such problems (p=0.008) openly. Years of study correlate significantly (p=0.043) with episodes of sleep paralysis represented in Table 2. A generous amount of second professional year (n=68, 59.1%) majorly experience moderate dreams. First (n=49, 90.7%) and final-year undergraduates (n=42, 91.3%) experienced the highest number of nightmares. However, sizeable incidences of nightmares were also chronicled in second (n=88, 76.5%), third (n=35, 81.4%), and fourth (n=32, 78%) years and were associated significantly (p=0.074). Second-year students (n=44, 38.3%) claimed to have a maximum number of episodes per night than others. A significant association was found (p=0.004) when data were compared with episodes of sleep paralysis from frist to final year. Second-year students (n=24, 33.3%) marked that they experienced one attack per month, while final-year students (n=1, 2.4%) hardly experienced one per week. Similar results were found when comparing personal experience, showing a meaningful connection. Continuing the trend, second-year students felt the maximum number of symptoms during paralytic episodes resulting again in substantial relation (p=0.085). Also, (n=47, 40.9%) second-year students started experiencing sleep paralysis between 15 and 20 years of age.

**Table 2:** Comparing sleep paralysis effect with professional years.

Questions	Response	1st year	2nd year	3rd year	4th year	5th year	p-Value
Have you ever experienced sleep paralysis?	Yes	37 (68.5%)	72 (63.7%)	34 (79.1%)	28 (68.3%)	21 (45.7%)	0.066
	No	17 (31.5%)	41 (36.2%)	9 (20.9%)	13 (31.7%)	25 (54.3%)	
If yes, how often?	At least one episode per week	4 (10.8%)	10 (13.8%)	6 (17.6%)	5 (17.8%)	3 (14.3%)	0.004
	More than one episode per week	14 (37.8%)	15 (20.8%)	8 (23.5%)	8 (28.5%)	2 (9.5%)	
	At least one episode per month	8 (21.6%)	24 (33.3%)	4 (11.7%)	5 (17.8%)	6 (28.5%)	
	More than one episode per month	7 (18.9%)	6 (8.3%)	6 (17.6%)	2 (7.1%)	3 (14.3%)	
	At least one episode per year	4 (10.8%)	17 (23.6%)	10 (29.4%)	8 (28.5%)	7 (33.3%)	
What are the causes of sleep paralysis?	Witch crafting	28 (52.8%)	46 (40%)	15 (34.9%)	15 (36.6%)	25 (54.3%)	0.034
	Drug abuse	35 (66.0%)	72 (62.6%)	22 (51.2%)	21 (51.2%)	28 (60.9%)	
	Nightmares	16 (29.6%)	27 (23.5%)	13 (30.2%)	16 (39.0%)	11 (23.9%)	
	Dying dreams	38 (70.3%)	96 (83.5%)	32 (74.4%)	24 (58.5%)	34 (73.9%)	
	Paranormal activities	9 (16.9%)	68 (59.1%)	12 (27.9%)	14 (34.1%)	18 (39.1%)	
	Flying dreams	12 (22.2%)	60 (52.2%)	14 (32.6%)	13 (31.7%)	25 (54.3%)	
	Hypnopompic and hypnagogic imaginations	39 (72.2%)	59 (51.3%)	27 (62.8%)	28 (68.3%)	38 (82.6%)	
Do you think people with sleep paralysis are reluctant to talk about this topic?	Yes	26 (50.0%)	53 (46.1%)	21 (48.8%)	21 (51.2%)	24 (52.2%)	<0.001
	No	28 (51.8%)	62 (53.9%)	22 (51.2%)	20 (48.8%)	22 (47.8%)	
Do you think sleep paralysis leads to anxiety?	Yes	44 (81.4%)	87 (75.7%)	30 (69.8%)	29 (70.7%)	35 (76.1%)	0.007
	No	10 (19.2%)	28 (24.3%)	13 (30.2%)	12 (29.3%)	10 (21.7%)	
What did you experience during your sleep paralytic episodes?	Inability to move	32 (61.5%)	52 (45.2%)	26 (60.5%)	15 (36.6%)	14 (20.4%)	0.085
	Inability to speak	33 (63.5%)	54 (47.0%)	26 (60.5%)	19 (46.3%)	14 (30.4%)	
	Sense of suffocation	11 (21.2%)	36 (31.3%)	14 (32.6%)	9 (22.0%)	11 (23.9%)	
	Unusual auditory or visual sensation	32 (61.5%)	37 (32.2%)	25 (58.1%)	18 (43.9%)	13 (28.3%)	
	Attack preceded by terrifying dream or thought	18 (34.6%)	21 (18.3%)	16 (37.2%)	11 (26.8%)	7 (15.2%)	
Do you have a family history or experience any of these conditions?	Automatic behavior	3 (5.8%)	2 (1.7%)	0 (0.0%)	1 (2.4%)	0 (0.0%)	0.045
	Bipolar disorder	5 (9.6%)	6 (5.2%)	1 (2.3%)	2 (4.9%)	1 (2.2%)	
	Hallucination	1 (1.9%)	1 (0.9%)	1 (2.3%)	2 (4.9%)	1 (2.2%)	
	Non-restorative sleep	4 (7.7%)	5 (4.3%)	2 (4.7%)	0 (0.0%)	2 (4.3%)	
	Nocturnal leg cramps	7 (13.5%)	8 (7.0%)	6 (14.0%)	5 (12.2%)	4 (8.7%)	
	Sleep apnea	1 (1.9%)	1 (0.9%)	1 (2.3%)	1 (2.4%)	0 (0.0%)	
	Insomnia	7 (13.5%)	16 (13.9%)	5 (11.6%)	6 (14.6%)	2 (4.3%)	
	Narcolepsy	3 (5.8%)	2 (1.7%)	1 (2.3%)	2 (4.9%)	1 (2.2%)	
	None	21 (40.4%)	74 (64.3%)	26 (60.5%)	22 (53.7%)	35 (76.1%)	
How many hours do you sleep during weekdays?	4 h	4 (7.7%)	8 (7.0%)	5 (11.6%)	1 (2.4%)	4 (8.7%)	0.025
	6 h	25 (47.1%)	65 (57.5%)	21 (48.8%)	24 (58.5%)	13 (28.3%)	
	8 h	20 (38.5%)	33 (28.7%)	15 (34.9%)	11 (26.8%)	20 (43.5%)	
	10 h	5 (9.4%)	6 (5.2%)	2 (4.7%)	3 (7.3%)	3 (6.5%)	
	12 h	0 (0.0%)	1 (0.9%)	0 (0.0%)	2 (4.9%)	6 (13.0%)	
How many hours do you sleep during weekend?	4 h	1 (1.8%)	1 (0.9%)	1 (2.3%)	1 (2.4%)	1 (2.2%)	0.036
	6 h	3 (5.5%)	8 (7.0%)	2 (4.7%)	1 (2.4%)	2 (4.3%)	
	8 h	18 (33.3%)	42 (37.1%)	13 (30.2%)	19 (46.3%)	16 (34.8%)	
	10 h	24 (44.4%)	47 (40.9%)	23 (53.5%)	13 (31.7%)	16 (34.8%)	
	12 h	8 (14.8%)	15 (13.0%)	4 (9.3%)	7 (17.1%)	11 (23.9%)	
How many hours of sleep do you require to feel fresh?	4 h	3 (5.8%)	10 (8.7%)	1 (2.3%)	0 (0.0%)	1 (2.2%)	0.047
	6 h	11 (21.2%)	22 (19.1%)	12 (27.9%)	2 (4.9%)	3 (6.5%)	
	8 h	25 (48.1%)	49 (43.3%)	22 (51.2%)	27 (65.9%)	25 (54.3%)	
	10 h	10 (18.5%)	21 (18.3%)	5 (11.6%)	11 (26.8%)	13 (28.3%)	
	12 h	5 (9.6%)	11 (9.6%)	3 (7.0%)	1 (2.4%)	4 (8.7%)	
Do you experience any paralytic episodes during your daytime sleep?	Yes	13 (24.0%)	18 (16.0%)	6 (14.0%)	7 (17.1%)	2 (4.3%)	0.088
	No	41 (75.9%)	95 (84.0%)	37 (86.0%)	34 (82.9%)	44 (95.7%)	
At what age did you first experience sleep paralysis	Less than 10 years	0 (0.0%)	9 (12.5%)	4 (9.3%)	0 (0.0%)	0 (0.0%)	0.083
	11-14 years	5 (9.6%)	16 (13.9%)	8 (18.6%)	4 (9.8%)	5 (10.9%)	
	15-20 years	29 (55.8%)	47 (40.9%)	17 (39.5%)	21 (51.2%)	11 (23.9%)	
	>20 years	4 (10.8%)	0 (0.0%)	4 (9.3%)	2 (4.9%)	4 (8.7%)	
	>25 year	0 (0.0%)	0 (0.0%)	1 (2.3%)	1 (2.4%)	1 (4.7%)	
	>30 years	0 (0.0%)	0 (0.0%)	0 (0.0%)	0 (0.0%)	0 (0.0%)	
At what time do you experience sleep paralysis?	Beginning of sleep	6 (11.5%)	6 (5.2%)	3 (7.0%)	1 (2.4%)	3 (6.5%)	0.015
	Middle of the night	17 (32.7%)	37 (32.2%)	22 (51.2%)	11 (26.8%)	10 (21.7%)	
	End of sleep	7 (13.5%)	18 (15.7%)	5 (11.6%)	8 (19.5%)	2 (4.3%)	
	More prevalent during the first 2 hours after sleep onset	4 (14.8%)	10 (8.7%)	4 (9.3%)	6 (14.6%)	4 (8.7%)	
	At sleep offset	3 (11.1%)	1 (1.4%)	0 (0.0%)	2 (4.9%)	2 (9.5%)	
Do you use mobile phone before bedtime?	Yes	51 (94.4%)	108 (95.6%)	41 (95.3%)	37 (90.2%)	42 (91.3%)	0.067
	No	3 (5.8%)	5 (4.4%)	2 (4.7%)	4 (9.8%)	4 (8.7%)	
What kind of online content do you use before bedtime?	Online magazines	1 (1.9%)	1 (0.9%)	1 (2.3%)	0 (0.0%)	0 (0.0%)	0.088
	E-commerce	0 (0.0%)	1 (0.9%)	0 (0.0%)	0 (0.0%)	1 (2.2%)	
	Blogs	1 (1.9%)	3 (2.6%)	1 (2.3%)	2 (4.9%)	3 (6.5%)	
	Portfolio	0 (0.0%)	2 (1.7%)	1 (2.3%)	0 (0.0%)	1 (2.2%)	
	Social media	50 (96.1%)	108 (93.9%)	40 (93.0%)	38 (92.7%)	39 (84.8%)	
What was your sleeping position when you experience sleep paralysis?	Supine	21 (56.7%)	37 (51.3%)	18 (52.9%)	18 (64.2%)	11 (50.0%)	0.093
	Prone	2 (5.4%)	7 (9.7%)	5 (14.7%)	2 (7.1%)	2 (9.0%)	
	Left-side	10 (27.0%)	14 (19.4%)	3 (8.8%)	4 (14.2%)	3 (14.3%)	
	Right-side	4 (10.8%)	14 (19.4%)	8 (23.5%)	4 (14.2%)	5 (22.7%)	
What type of dream you usually experience?	Peaceful	2 (3.8%)	10 (8.7%)	2 (4.7%)	3 (7.3%)	5 (10.9%)	0.043
	Disturbed	16 (29.6%)	21 (18.3%)	13 (30.2%)	11 (26.8%)	10 (21.7%)	
	Moderate	32 (59.2%)	68 (59.1%)	22 (51.2%)	22 (53.7%)	25 (54.3%)	
	None	4 (7.4%)	16 (13.9%)	6 (14.0%)	5 (12.2%)	6 (13.0%)	
Have you experienced nightmares?	Yes	49 (90.7%)	88 (76.5%)	35 (81.4%)	32 (78.0%)	42 (91.3%)	0.074
	No	5 (9.3%)	27 (23.5%)	8 (18.6%)	9 (22.0%)	4 (8.7%)	
If yes, how often?	Always	5 (9.2%)	5 (4.3%)	3 (7.0%)	3 (7.3%)	0 (0.0%)	0.017
	Sometime	20 (37.0%)	44 (38.3%)	16 (37.2%)	12 (29.3%)	21 (45.7%)	
	Infrequently	8 (14.8%)	16 (13.9%)	7 (16.3%)	7 (17.1%)	8 (17.4%)	
	Rarely	16 (29.6%)	23 (20.0%)	9 (20.9%)	10 (31.2%)	13 (28.3%)	
Were you always in a state of stress during your sleep paralysis episodes?	Yes	21 (40.4%)	35 (30.4%)	13 (30.2%)	12 (29.3%)	11 (23.9%)	0.004
	No	15 (28.8%)	38 (33.0%)	21 (48.8%)	16 (39.0%)	10 (21.7%)	
If yes, what is the reason of your stress?	Financial problem	0 (0.0%)	2 (1.7%)	1 (2.3%)	0 (0.0%)	0 (0.0%)	0.078
	Academic burden	12 (23.1%)	20 (17.4%)	6 (14.0%)	8 (19.5%)	8 (17.4%)	
	Any traumatic experience	6 (11.5%)	7 (6.1%)	5 (11.6%)	2 (4.9%)	2 (4.3%)	
Do you think sleep paralysis has relation with your life events?	Yes	23 (44.2%)	41 (35.7%)	13 (30.2%)	13 (31.7%)	9 (19.6%)	0.056
	No	12 (23.1%)	32 (27.8%)	21 (48.8%)	14 (34.1%)	12 (26.1%)	
Do you think sleep paralysis episodes affect your academics?	Yes	14 (26.9%)	25 (21.7%)	8 (18.6%)	7 (17.1%)	9 (19.6%)	0.042
	No	23 (44.2%)	48 (41.7%)	26 (60.5%)	21 (51.2%)	12 (26.1%)	

Additionally, a significant upshot (p=0.045) was renowned from first to final-year students when questioned for any psychotic/sleep paralysis family history. On the other hand, hours of sleep during weekdays (p=0.025) and weekends (p=0.036) show homogenous magnitudes. Nevertheless, hours of sleep needed to feel fresh show a remarkable bond (p=0.047) with the year of study. Virtually, students of all years decide on 8 hours of sleep. A moderately equal population was found to be in courtesy of either 6 or 10 hours as indispensable sleeping hours. Sleep paralysis in the middle of the night was found among students in second year (n=37, 32.2%), proposing an imperative understanding (p=0.015). Barely any student (n=97, 84.3%) brings about daytime sleep paralysis setting up an essential connection (p=0.088).

Mobile phone usage parade inconsequentially (p=0.067) when compared. All year students used cell phones right before bedtime. Second year students (n=108, 95.6%) were on the top followed by third (n=41, 95.3) and first years (n=51, 94.4) students. Like the previous result, the type of content watched before bedtime illustrates a substantial relation when likened (p=0.088). Sleep position while experiencing paralytic episode inaugurate paltry (p=0.093) assessing supine position as communal. In contrast, sleep paralysis significantly relates to (p=0.056) past life events coupled with the financial crisis, illustrating trivial nexus (p=0.078). Likewise, the majority disagree on the bad effect of paralytic episodes on academics (p=0.042), while anxiety and depression do (p=0.007). They are shown in Table 2.

In Table 3, moderate dreams were found majorly in the range of 19-21 years, linking it trivially (p=0.097). Additionally, the uppermost age 22-24 years had experience nightmares numerous times (p=0.049) side to side with the highest episodes rate (p=0.087). Sleep paralysis was majorly prevalent in individuals of more than 24 years of age (p=0.076). It was the significant victims with episodes of one per week (p=0.008). Unusual auditory or visual sensations and inability to move and speak were also substantial concerns of the above 24-year age group (n=5, 100%), with a sense of suffocation being of most minor occurrence (p=0.069). Fifteen to 20 years of age were selected as the age of onset of sleep paralysis by all age groups (p=0.076), with a significant population denying any family history (p=0.037). Analysis of sleep routine on weekdays showed 6 hours as manageable time by the majority (p=0.027) while 10 hours on weekends (p=0.064) marked ominously. However, the hours required to feel fresh were chosen was 8 hours (p=0.060).

**Table 3:** Responses of undergraduate age groups with contributing factors of sleep paralysis.

Questions	Response	16-18	19-21	22-24	>24	p-Value
Have you ever experienced sleep paralysis?	Yes	17 (54.8%)	143 (70.4%)	28 (47.5%)	4 (100%)	0.007
	No	14 (45.2%)	60 (29.6%)	31 (52.5%)	0 (0.0%)	
If yes, how often?	At least one episode per week	2 (6.5%)	20 (13.9%)	6 (10%)	3 (75%)	0.008
	More than one episode per week	5 (16.2%)	39 (27.2%)	4 (6.7%)	0 (0.0%)	
	At least one episode per month	3 (9.7%)	37 (18.2%)	5 (8.3%)	1 (25%)	
	More than one episode per month	2 (6.4%)	16 (11.1%)	3(5%)	0 (0.0%)	
	At least one episode per year	5 (29.4%)	31 (15.3%)	10 (16.7%)	0 (0.0%)	
What are the causes of sleep paralysis?	Witch crafting	12 (38.7%)	87 (42.9%)	30 (50.0%)	0 (0.0%)	0.074
	Drug abuse	17 (54.8%)	123 (60.6%)	36 (60%)	2 (40%)	
	Nightmares	9 (29.0%)	58 (28.6%)	11 (18.3%)	5 (100%)	
	Dying dreams	22 (71.0%)	155 (76.45)	44 (73.3%)	3 (60%)	
	Paranormal activities	10 (32.3%)	88 (43.3%)	23 (38.3%)	0 (0.0%)	
	Flying dreams	11 (35.5%)	82 (40.4%)	28 (46.7%)	3 (60.0%)	
	Hypnopompic imaginations	22 (71%)	119 (58.6%)	45 (75%)	5 (100%)	
Do you think people with sleep paralysis are reluctant to talk about this topic?	Yes	14 (45.2%)	99 (48.8%)	30 (50%)	2 (40%)	0.078
	No	17 (54.8%)	104 (51.2%)	30 (50%)	3 (60%)	
Do you think sleep paralysis leads to anxiety?	Yes	24 (77.4%)	148 (72.9%)	48 (80%)	4 (100%)	0.005
	No	7(22.6)	55 (27.1%)	11 (18.3%)	0(0.0%)	
What did you experience during your sleep paralytic episodes?	Inability to move	13 (41.9%)	103 (50.7%)	18 (30%)	5 (100%)	0.069
	Inability to speak	13 (41.9%)	109 (53.7%)	19 (31.7%)	5 (100%)	
	Sense of suffocation	5 (16.1%)	64 (31.5%)	12 (20%)	0 (0.0%)	
	Unusual auditory or visual sensation	13 (41.9%)	88 (43.3%)	19 (31.7%)	5 (100%)	
	Attack preceded by terrifying dream or thought	6 (19.4%)	51 (25.1%)	12 (20%)	4 (80%)	
Do you have a family history or experience any of these conditions?	Automatic behavior	0 (0.0%)	6(3%)	0 (0.0%)	0 (0.0%0	0.037
	Bipolar disorder	4 (12.9%)	10 (4.9%)	1 (1.7%)	1 (20%)	
	hallucination	0 (0.0%)	4(2%)	1 (1.7%)	0 (0.0%)	
	Non-restorative sleep	2 (6.5%)	8 (3.9%)	2 (3.3%)	1 (20%)	
	Nocturnal leg cramps	2 (6.5%)	20 (9.9%)	8 (13.3%)	0 (0.0%)	
	Sleep apnea	0 (0.0%)	3 (1.5%)	1 (1.7%)	0 (0.0%)	
	Insomnia	7 (22.6%)	24 (11.8%)	5 (8.4%)	1 (20%)	
	Narcolepsy	2 (6.5%)	5 (2.5%)	2 (3.3%)	0 (0.0%)	
	None	14 (45.2%)	122 (60.1%)	40 (66.7%)	2 (40%)	
How many hours do you sleep during weekdays?	4 h	3 (9.7%)	17 (8.4%)	2 (3.3%)	0 (0.0%)	0.027
	6 h	14 (45.2%)	107 (52.7%)	26 (43.3%)	2 (28.5%)	
	8 h	10 (32.3%)	65 (32.0%)	23 (38.3%)	2 (28.5%)	
	10 h	4 (12.9%)	11 (5.4%)	4 (6.7%)	0 (0.0%)	
	12 h	0 (0.0%)	1 (0.5%)	4 (6.7%)	2 (28.5%)	
How many hours do you sleep during weekend?	4 h	0 (0.0%)	4 (2%)	1 (1.7%)	0 (0.0%)	0.064
	6 h	1 (3.2%)	11 (5.4%)	3(5%)	1 (20%)	
	8 h	8 (25.8%)	77 (37.9%)	21 (35.5%)	2 (40%)	
	10 h	16 (51.6%)	87 (42.9%)	20 (33.3%)	1 (20%)	
	12 h	6 (19.4%)	24 (11.8%)	13 (21.7%)	1 (20%)	
How many hours of sleep do you require to feel fresh?	4 h	2 (6.5%)	11 (5.4%)	2 (3.3%)	0 (0.0%)	0.060
	6 h	3 (9.7%)	42 (20.7%)	5 (8.3%)	0 (0.0%)	
	8 h	16 (51.6%)	95 (46.8%)	33 (55.9%)	4 (80%)	
	10 h	8 (25.8%)	36 (17.7%)	16 (26.7%)	0 (0.0%)	
	12 h	2 (6.5%)	19 (9.4%)	2 (3.3%)	1 (20%)	
Do you experience any paralytic episodes during your daytime sleep?	Yes	6 (19.4%)	35 (17.2%)	2(3.3%)	1 (20%)	0.086
	No	25 (80.6%)	167 (82.3%)	57 (95%)	4 (80%)	
At what age did you first experience sleep paralysis?	Less than 10 years	0 (0.0%)	11 (5.4%)	1 (1.7%)	0 (0.0%)	0.076
	11-14 years	4 (12.9%)	29 (14.3%)	5 (8.3%)	0 (0.0%)	
	15-20 years	12 (38.7%)	95 (46.8%)	17 (28.3%)	1 (20%)	
	>20 years	1 (3.2%)	4 (2%)	5 (8.3%)	2 (40%)	
	>25 year	0 (0.0%)	1 (0.5%)	0 (0.0%)	1 (20%)	
	>30 years	0 (0.0%)	1 (0.5%)	0 (0.0%)	1 (20%)	
At what time do you experience sleep paralysis?	Beginning of sleep	2 (6.5%)	13 (6.4%)	3(5%)	1 (20%)	0.007
	Middle of the night	9 (29%)	74 (36.5%)	11 (18.3%)	3 (60%)	
	End of sleep	2 (6.5)	31 (15.3%)	7 (11.7%)	0 (0.0%)	
	More prevalent during the first 2 hours after sleep onset	2 (6.5%)	18 (8.9%)	6 (10%)	0 (0.0%)	
	At sleep offset	1 (3.2%)	5 (2.5%)	0 0.0%)	1 (20%)	
Do you use mobile phone before bedtime?	Yes	30 (96.8%)	191 (94.1%)	56 (93.3%)	3 (60%)	0.004
	No	1 (3.2%)	12 (5.9%)	2 (3.3%)	2 (40%)	
What kind of online content do you use before bedtime?	Online magazines	0 (0.0%)	3 (1.5%)	0 (0.0%)	0 (0.0%)	0.068
	E-commerce	0 (0.0%)	1 (0.5%)	1 (1.7%)	0 (0.0%)	
	Blogs	0 (0.0%)	6(3%)	3 (5%)	1 (20%)	
	Portfolio	0 (0.0%)	4 (2%)	0 (0.0%)	0 (0.0%)	
	Social media	31 (100%)	185 (91.1%)	55 (91.7%)	4 (80%)	
What was your sleeping position when you experience sleep paralysis?	Supine	14 (45.2%)	72 (35.5%)	19 (31.7%)	0 (0.0%)	0.086
	Prone	0 (0.0%)	21 (13.7%)	3 (5%)	0 (0.0%)	
	Left-side	1 (3.2%)	30 (19.6%)	1 (1.7%)	2 (50%)	
	Right-side	2 (6.5%)	20 (12.3%)	5 (17.8%)	2 (50%)	
What type of dream you usually experience?	Peaceful	1 (3.2%)	13 (6.4%)	8 (13.3)	0 (0.0%)	0.097
	Disturbed	8 (25.8%)	48 (23.6%)	13 (21.7%)	2 (40%)	
	Moderate	17 (54.8%)	121 (59.6%)	29 (48.3%)	2 (40%)	
	None	5 (16.1%)	21 (10.3%)	9 (15.2%)	0 (0%)	
Have you experienced nightmares?	Yes	26 (83.9%)	163 (80.3%)	53 (89.8%)	4 (80%)	0.049
	No	5 (16.1%)	40 (19.7%)	6 (10.2%)	0 (0%)	
If yes, how often?	Always	3 (9.7%)	12 (5.9%)	1 (1.7%)	0 (0.0%)	0.087
	Sometime	11 (35.5%)	75 (36.9%)	25 (41.7%)	2 (40%)	
	Infrequently	5 (16.1%)	30 (14.8%)	10 (16.7%)	1 (20%)	
	Rarely	7 (22.6%)	46 (28.2%)	17 (28.3%)	1 (20%)	
Were you always in a state of stress during your sleep paralysis episodes?	Yes	5 (16.1%)	67 (33%)	17 (28.3%)	3 (60%)	0.038
	No	12 (38.7%)	75 (36.9%)	11 (18.3%)	2 (40%)	
If yes, what is the reason of your stress?	Financial problem	0 (0.0%)	3 (1.5%)	0 (0.0%)	0 (0.0%)	0.076
	Academic burden	4 (12.9%)	42 (20.7%)	7 (11.7%)	1 (20%)	
	Any traumatic experience	1 (3.2%)	13 (6.4%)	7 (11.7%)	1 (20%)	
Do you think sleep paralysis has relation with your life events?	Yes	8 (25.8%)	75 (36.9%)	12 (20%)	4 (80%)	0.038
	No	8 (25.8%)	66 (32.5%)	16 (26.7%)	1 (20%)	
Do you think sleep paralysis episodes affect your academics?	Yes	5 (16.1%)	48 (23.6%)	7 (11.7%)	3 (60%)	0.056
	No	12 (38.7%)	95 (46.8%)	21 (35%)	2 (40%)	

Apart from that, the association between sleep paralysis and time of occurrence was found to be majorly in the middle of the night (p=0.007). Daytime paralysis was also recorded (p=0.086). A significant preponderance of mobile usage was found in ages 16-18 years (p=0.004), and most were exposed to social media right before bedtime (p=0.068). Sleeping positions were also assessed, and the supine position was dominant between all age groups (p=0.086). Out of other probable causes, nightmares and hypnopompic imaginations outnumbered in percentage (n=5, 100%) chosen by age group 24 and above (p=0.074), as shown in Table 3.

A tight connection between sleep paralysis and stress was evaluated (p=0.038), and the academic burden was chosen as a principal source by age group 19-21 years (p=0.076), as shown in Table 3. The relation between sleep paralysis and life events was agreed upon (p=0.038), while its connection with affecting academics suffered disagreement (p=0.056). A profound association in Table 3 was found between sleep paralysis and anxiety and depression (p=0.005), while approximately equal responses were reported for those in favor and disagreement in reluctance to talk about this topic openly (p=0.078). The comparison of age with the age of the first onset of paralytic episodes using ANOVA displayed an insignificant association (p=0.174). When age was co-related to sleep routine, weekdays showed a significant association (p=0.040), while weekends presented a minimal relation (p=0.081). Furthermore, a highly insignificant connection between age and self-rated internet usage was found (p=0.345). Moreover, the comparison of age with both gender using the t-test and year of the study exhibited insignificant association (p=0.192).

## Discussion

According to the literature reviewed, episodes of sleep paralysis are relatively common among the population. When inquired about the source of information, the response of partakers was diverse. Sharpless and Kliková reported in their study that at least one episode of sleep paralysis had been experienced in 8% general population, 28% of students, and 32% of psychiatric patients [10,11]. In contrast, our results frequency was quite different throughout the year, but the most common was one episode per month. In our study, sleep paralysis was found to be more common in women rather than in men. In another study, sleep paralysis was widespread among medical students, but the cause was unknown. Lišková et al. reported in a comparative study that sleep-paralytic episodes are related to nightmares. As its frequency increases, sleep paralysis episode rises. A total of 69% of participants reported that they experienced intense or extreme fear in their attack of sleep paralysis, but 16% stated that they experienced a pleasant feeling [12].

Additionally, Denis et al., in their analysis, showed that sleep paralysis has an independent relation with sleep quality, anxiety, and exposure to threatening events [13]. However, in our study, dying dreams were a prevalent cause that resulted in sleep paralysis. Likewise, in our research, the inability to speak was the most common experience. They also underwent unusual auditory and visual sensations. On the contrary, a study by Sharpless and Kliková found that half of the participants experienced delusion that someone was present in the room [10]. However, less than 25% felt a visual hallucination of a human or non-human being [14].

On the other hand, a study by Hufford found multiple reports of hallucinated, wondrous non-human entities explained by a paranormal experience [11,15]. In his research, Fukuda et al. found that many students assumed sleep paralysis as some dream [12]. Several Japanese people believed that this strange phenomenon of sleep paralysis was related to some supernatural power or evil spirits. This spiritual idea must have come into the people because of the hallucinatory reality of sleep-paralytic episodes [16].

A report by Denis et al. unveiled that the frequency of sleep paralysis was shown to have an independent association with the age of occurrence [13,17]. Their results were consistent with our study as we found different age values and their occurrence of sleep paralysis. However, the maximum occurrence age was 15-20 years. Another survey by Lišková et al. reported that the mean age of their participants to have the first episode of sleep paralysis was 17 years; however, 24% of respondents had their first paralytic episode before the age of 15 years [4,18]. In our study, a very diverse pattern was found among the respondents when evaluating the occurrence of sleep paralysis throughout the night. Some stated it was more prevalent during the first 2 hours of sleep. Most of our responses were from the middle of the night; some respondents also said they had experienced the episode even at the end of sleep. Girard and Cheyne reported a similar study as ours, as their respondents mainly experienced it during the first 2 hours of sleep [14,19]. Plus, Spanos et al. stated that sleep onset is more common than others [15,20]. In another study, sleep offset was also reported [21]. While Cheyne stated in his research that the timing of sleep paralysis was a combination of the beginning, the middle of the night, and the end of the night [2,22].

Sleep routines among medical students have always been versatile. Most of them could not get even sleep for 7 hours. A study reported a similar practice that only 24% of university students get sleep of 7 hours in the United Kingdom [21], 30% in Korea [22], and 49% in Taiwan [23]. Moreover, a Lithuanian Survey reported that 5-hour sleep by more than half of the university students [22]. Our study demonstrated the abovementioned reports that many respondents get 6 hours of sleep. The ratio of academic burden has always been high for medical students as this is constant in a medical student's life. This burden must have to influence sleep quality and sleep routine. When inquired about the topic's sensitivity, a study concluded by Bahammam et al. related that decreased sleep at night plus late bedtimes during weekdays and increased daytime sleepiness have a negative association with academic performance in medical students [22]. A similar Brazilian study showed a relationship between academics and sleep length, onset, and regularity [23].

In contrast, the responses from our study disprove the above statement as it shows that only a few candidates agree that sleep paralysis significantly impacts their academic life. Several studies prove that poor sleep routine affects sleep quality. Sleep quality is a significant factor in determining different aspects of an individual's daily routine habits. Curcio et al. in 2006 unveiled that sleep quality is related to learning and academic performance [24]. This was shown in a study conducted in China that stated that the people who rated their sleep quality as very good showed less frequency of sleep paralysis episodes compared with those who placed their sleep to be wrong and had a higher chance of such attacks [25]. A consistent result was found in a Japanese study that collected a record of 90,000 participants and had similar results [26].

It was apparent in research by Young et al. that sleep paralysis correspondence with stress related to traumatic events [27]. The analysis by Denis also indicates the relationship between increased traumatic events and sleep paralysis [13]. Stress is vital in sleep paralytic episodes, as about half of our participants replied affirmatively.

Limitations

The limitation of our study is that we randomly recruited participants with an age limit between 18-30 years. However, our collected data does not concede the upper age limit. Therefore, this study only corresponds to the mental aspects of middle-aged people related to any medical field. Secondly, the data was collected from all medical students only, which can also be considered a limitation.

Further research is required to evaluate sleep paralysis in every aspect and to be explained in detail. Future research must target every age group, including the young, middle, and older populations. Moreover, we suggest that future research on this topic include participants with episodes of sleep paralysis in addition to the general public as the sample population. Depth and awareness of this condition among the general population will hopefully lead to knowing the cause, as different aspects of different respondents may lead to the actual cause.

## Conclusions

Sleep paralysis in medical students, despite affecting a significant population, is still controversial. The reasons for its occurrence are deeply rooted in either a traumatic incident or any prevailing stress. Whatever the cause, owing to its academic and social effects, it is necessary to address this problem on a larger scale in all medical universities. For individuals prone to its occurrence at an unusually higher rate, there is a desperate need for rehabilitation and psychiatric care. Doctors and psychiatrists must be trained to develop a deep doctor-patient relationship to curb this issue. An intervention by the health system is required. Awareness must be held with the aim and objective of addressing such problems prevailing in the medical profession and devising a way to tackle such incidences.
